# Caloric restriction promotes cell survival in a mouse model of normal tension glaucoma

**DOI:** 10.1038/srep33950

**Published:** 2016-09-27

**Authors:** Xiaoli Guo, Atsuko Kimura, Yuriko Azuchi, Goichi Akiyama, Takahiko Noro, Chikako Harada, Kazuhiko Namekata, Takayuki Harada

**Affiliations:** 1 Visual Research Project, Tokyo Metropolitan Institute of Medical Science, Tokyo, Japan

## Abstract

Glaucoma is characterized by progressive degeneration of retinal ganglion cells (RGCs) and their axons. We previously reported that loss of glutamate transporters (EAAC1 or GLAST) in mice leads to RGC degeneration that is similar to normal tension glaucoma and these animal models are useful in examining potential therapeutic strategies. Caloric restriction has been reported to increase longevity and has potential benefits in injury and disease. Here we investigated the effects of every-other-day fasting (EODF), a form of caloric restriction, on glaucomatous pathology in EAAC1^−/−^ mice. EODF suppressed RGC death and retinal degeneration without altering intraocular pressure. Moreover, visual impairment was ameliorated with EODF, indicating the functional significance of the neuroprotective effect of EODF. Several mechanisms associated with this neuroprotection were explored. We found that EODF upregulated blood β-hydroxybutyrate levels and increased histone acetylation in the retina. Furthermore, it elevated retinal mRNA expression levels of neurotrophic factors and catalase, whereas it decreased oxidative stress levels in the retina. Our findings suggest that EODF, a safe, non-invasive, and low-cost treatment, may be available for glaucoma therapy.

Glaucoma is one of the leading causes of vision loss in the world and it is estimated that this condition will affect more than 80 million individuals worldwide by 2020, with at least 6.8 million individuals becoming bilaterally blind^1^. Glaucoma is characterized by progressive degeneration of retinal ganglion cells (RGCs) and their axons. Whisle this is usually associated with elevated intraocular pressure (IOP), there is a subset of glaucoma termed normal tension glaucoma (NTG) that presents with statistically normal IOP. There are several animal models of glaucoma with high IOP, including DBA/2J mice, an animal model that recapitulates the slow and progressive nature of human glaucoma^2^,^3^ and inducible models such as cauterization of episcleral veins^4^. In addition, we previously reported that loss of glutamate transporters (EAAC1 or GLAST) in mice leads to RGC degeneration that is similar to NTG and these animal models have been useful in examining potential therapeutic targets^5^,^6^,^7^,^8^,^9^,^10^.

Caloric restriction has been reported to increase longevity and has some benefits in injury and disease^11^. Every-other-day fasting (EODF) is a form of caloric restriction and it has been shown to exert neuroprotective effects when being implemented after rat cervical spinal cord injury^12^. EODF preserved neuronal integrity, dramatically reduced lesion volume by >50%, and increased sprouting of corticospinal axons^12^. There are multiple pathways known to be affected by EODF. For example, blood β-hydroxybutyrate (β-HB) levels, a ketone known to possess neuroprotective properties and recently reported to be an endogenous histone deacetylase (HDAC) inhibitor^13^, were increased by 2–3 fold on the fasting days^12^,^14^. β-HB has been shown to protect neurons in models of Alzheimer’s and Parkinson’s disease^15^ and ketogenic diet (KD) reduced cortical contusion volume after traumatic brain injury^16^. Moreover, EODF increased antioxidants and decreased oxidative damage in the brain by reducing ROS^17^. EODF also increased the expression levels of brain-derived neurotrophic factor (BDNF) in the brain^18^. In addition, EODF showed a direct effect on the cell death pathways by increasing anti-apoptotic proteins such as apoptosis repressor with a caspase recruitment domain, which inhibits caspase-2 activity and attenuates cytochrome c release from the mitochondria^19^.

In this study, we investigated the effects of EODF on RGC protection in EAAC1^−/−^ mice, an animal model of NTG. We monitored changes in retinal morphology of mice with or without EODF using spectral-domain optical coherence tomography (SD-OCT), which permits noninvasive, longitudinal and quantitative assessment of retinal structures in living animals^7^,^8^,^9^,^10^,^20^,^21^,^22^. In addition, we measured visual function using multifocal electroretinography (mfERG) to evaluate functional effects of EODF^5^,^6^,^7^,^8^,^9^,^10^,^21^,^23^,^24^. We also explored possible mechanisms associated with EODF-mediated neuroprotection in EAAC1^−/−^ mice.

## Results

### Caloric restriction suppresses retinal degeneration in a mouse model of NTG

**Figure 1:**
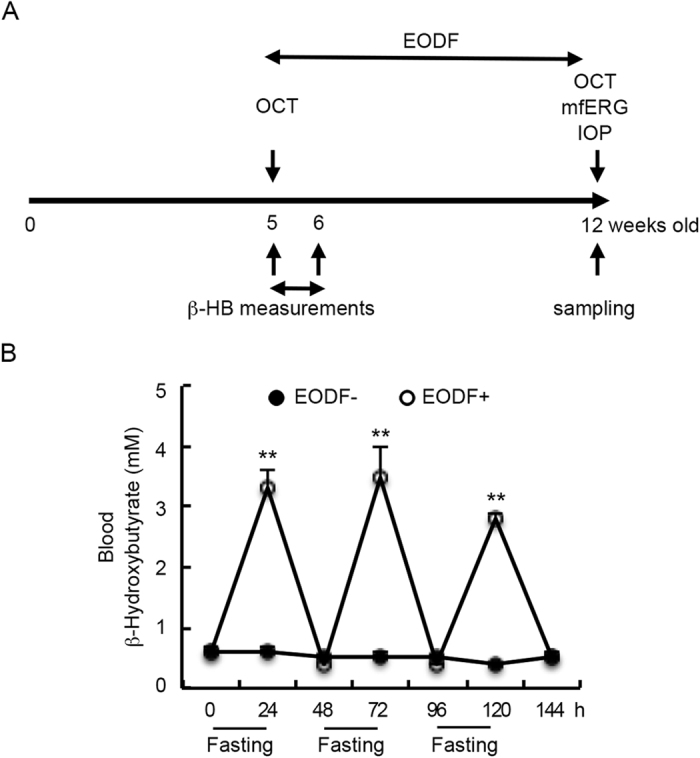
Experimental timeline and effects of EODF on blood β-HB levels in EAAC1^−/−^ mice. (**A**) Experimental protocols. EODF was started in EAAC1^−/−^ mice from 5 W and continued for 7 weeks. OCT, mfERG and IOP of mice were measured before sampling at 12 W. β-HB levels in the blood of mice were measured every 24 h in the first week of the experiment. The control group (EODF−) had continual access to food, while the EODF group (EODF+) had access to food on alternate days. Both groups were given ad libitum access to water. (**B**) Increased β-HB levels in the blood of EODF mice on fasting days. The data are presented as means ± SEM of six samples for each experiment. **P < 0.001.

The retinas of EAAC1^−/−^ mice show normal organization at 5 weeks old (5 W) and NTG-like retinal degeneration starts thereafter, as we previously reported^5^,^7^,^8^,^10^. To investigate whether caloric restriction is capable of ameliorating the NTG-like pathology in EAAC1^−/−^ mice, we performed EODF on EAAC1^−/−^ mice between 5 W and 12 W (Fig. 1A). Since caloric restriction has been reported to increase the β-HB concentration^12^, we first examined its concentration in the blood during EODF. The concentration of β-HB in EAAC1^−/−^ mice was significantly increased on the fasting days (Fig. 1B).

**Figure 2:**
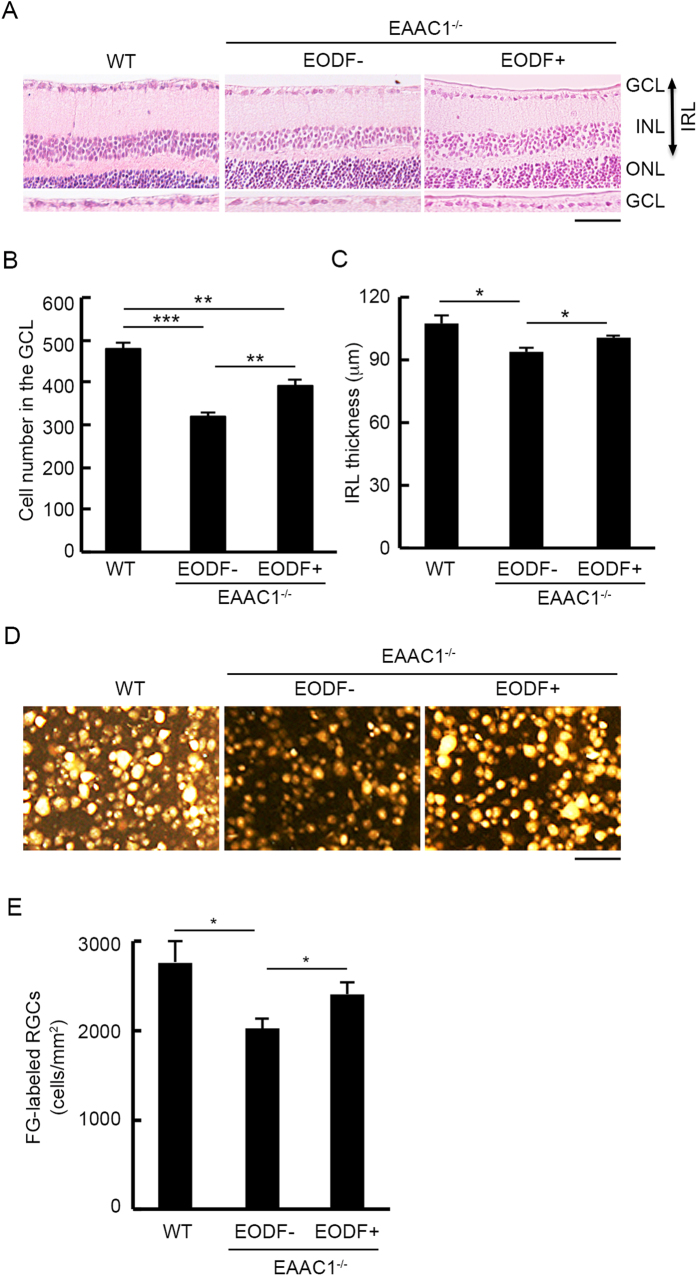
Effects of EODF on retinal degeneration in EAAC1^−/−^ mice. (**A**) Retinal sections stained with hematoxylin and eosin at 12 W in mice treated with or without EODF. Scale bar: 50 μm. GCL, ganglion cell layer; INL, inner nuclear layer; ONL, outer nuclear layer; IRL, inner retinal layer. (**B**,**C**) Quantitative analyses of the cell number in the GCL per section (**B**) and IRL thickness (**C**). (**D**) Representative images of FG-labeled RGCs at 12 W. Scale bar: 55 μm. (**E**) Quantitative analyses of RGCs in (**D**). Data are presented as means ± SEM of six samples for each experiment. ***P < 0.001, **P < 0.01, *P < 0.05.

We then examined the effects of EODF on retinal degeneration. The cell number in the ganglion cell layer (GCL) and the thickness of the inner retinal layer (IRL) were significantly decreased in EAAC1^−/−^ mice compared with wild type (WT) mice at 12 W (Fig. 2A–C). EODF significantly increased the number of surviving neurons in the GCL and IRL thickness compared with those in control mice (Fig. 2A–C). Because GCL contains cell types other than RGCs including displaced amacrine cells^25^, we next performed retrograde labeling of RGCs with Fluoro-Gold (FG) and determined the effect of EODF on RGC survival. Consistent with the results of cell counting in the GCL (Fig. 2B), the RGC number in EODF EAAC1^−/−^ mice was significantly increased compared with control EAAC1^−/−^ mice without EODF (Fig. 2D,E). These data demonstrate that EODF prevents RGC death observed in EAAC1^−/−^ mice.

**Figure 3:**
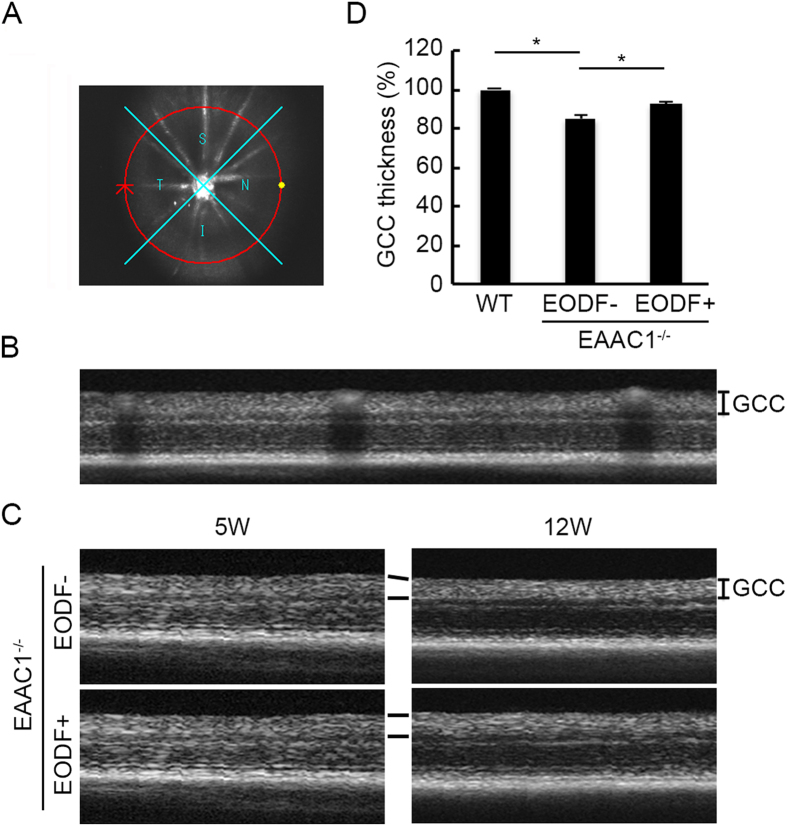
*In vivo* imaging of the retina in the control and EODF EAAC1^−/−^ mice. (**A**) An image of a scanning circle centering around the optic nerve disk. (**B**) Longitudinal evaluation of the GCC thickness by a circular scan. (**C**) OCT cross-sectional images of retinas at 5 W and 12 W in mice treated with or without EODF. GCC, ganglion cell complex. (**D**) Quantitative analyses of GCC thickness of control and EODF mice. The data are presented as means ± SEM of six samples for each experiment. *P < 0.05.

We also visualized retinal layers *in vivo* using SD-OCT^7^,^8^,^9^,^10^,^20^,^21^,^22^. The average thickness of the ganglion cell complex (GCC), which includes the nerve fiber layer, GCL and the inner plexiform layer, was measured by scanning the retina in a circle centering around the optic nerve disc (Fig. 3A,B). The average thickness of the GCC in the control EAAC1^−/−^ mice without EODF was significantly decreased compared with that in WT mice at 12 W, but such a reduction was significantly suppressed in EODF-treated EAAC1^−/−^ mice (Fig. 3C,D). These data indicate that EODF between 5 W and 12 W suppresses NTG-like retinal degeneration in EAAC1^−/−^ mice.

### Caloric restriction ameliorates visual impairment without affecting IOP

**Figure 4:**
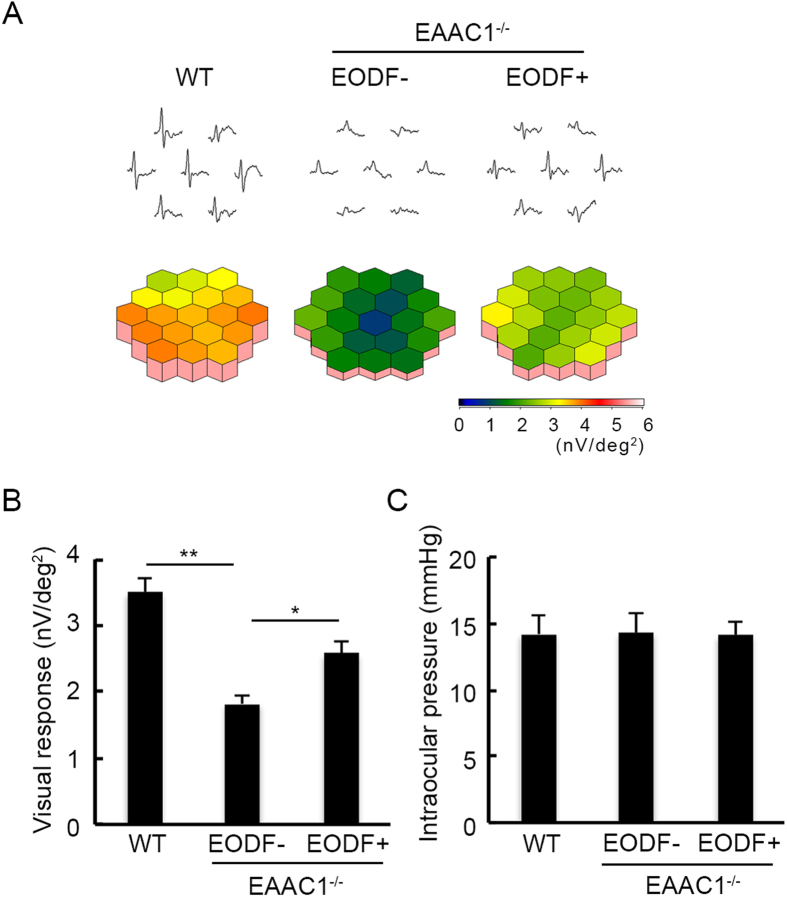
Effects of EODF on visual responses and IOP in EAAC1^−/−^ mice. (**A**) Averaged visual responses of the 2 K component demonstrated using three-dimensional plots. (**B**) Quantitative analysis of the visual response amplitude in (**A**). (**C**) Effects of EODF on IOP in EAAC1^−/−^ mice at 12 W. The data are presented as means ± SEM of six samples for each experiment. **P < 0.01, *P < 0.05.

In order to determine if the histological observation of EODF-mediated neuroprotection in EAAC1^−/−^ mice reflects functional aspects, we examined visual function using mfERG. We analyzed the second-order kernel (2 K) component, which appears to be a sensitive indicator of inner retinal dysfunction^5^,^6^,^7^,^8^,^9^,^10^,^21^,^23^,^24^ and is impaired in glaucoma patients^26^. The response topography demonstrating the 2 K component revealed that the average visual responses were impaired in all visual fields in EAAC1^−/−^ mice, but EODF treatment ameliorated the deterioration in visual function (Fig. 4A,B). These results verify that the neuroprotective effects of EODF on glaucomatous retinal degeneration in EAAC1^−/−^ mice are functionally significant.

We next examined the effects of caloric restriction on IOP. The IOP values of EODF-treated mice were not significantly altered compared to those of control mice (Fig. 4C). These results suggest that EODF prevents visual impairment associated with EAAC1^−/−^ mice and EODF-mediated neuroprotective effect is IOP-independent.

### EODF promotes histone acetylation and upregulates expressions of neurotrophic factors and an oxidative stress resistance gene in the retina

**Figure 5:**
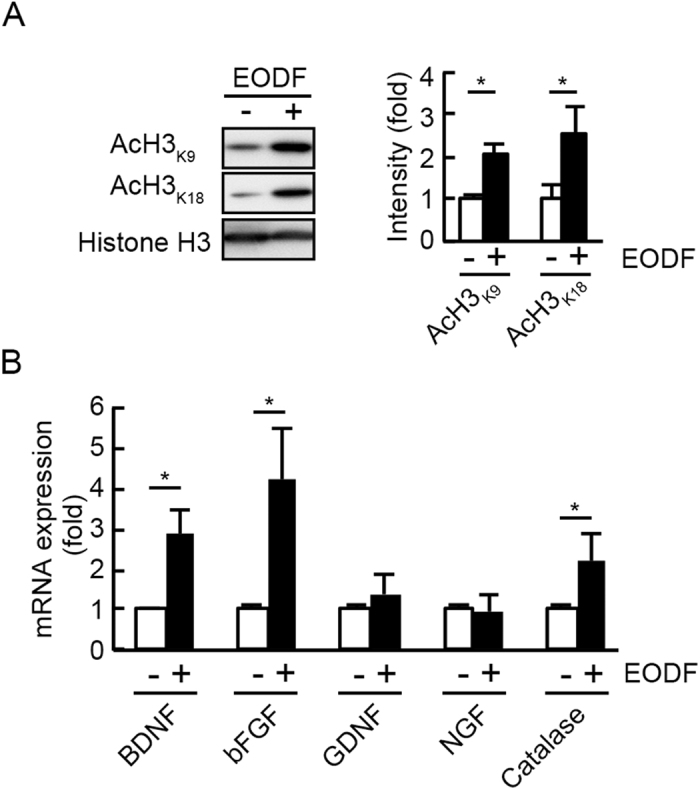
Effects of EODF on histone acetylation, neurotrophic factors and antioxidant gene expressions in the retina. (**A**) Expression levels of AcH3_K9_ and AcH3_K18_ in the retina of WT mice. Histone H3 was used as an internal control. (**B**) mRNA expression levels of neurotrophic factors (BDNF, bFGF, GDNF and NGF) and an oxidative stress resistance gene (catalase) were determined using quantitative PCR analyses. GAPDH was used as an internal control. The data are presented as means ± SEM of six samples for each experiment. *P < 0.05.

Next we investigated possible mechanisms associated with EODF-mediated neuroprotection. Since the concentration of β-HB, an endogenous HDAC inhibitor, was significantly increased upon EODF (Fig. 1B), we first investigated whether EODF could alter histone acetylation in the retina. To this aim, histones from retinas of WT mice undergone EODF for 7 days were purified. Acetylation of H3_K9_ and H3_K18_ was slightly detected in retinas of mice without EODF, and western blotting analysis revealed a significant upregulation of histone acetylation in retinas isolated from mice with EODF (Fig. 5A). Since acetylation of H3_K9_ influences various gene expressions^13^, EODF may alter gene expressions that result in neuroprotection. Therefore, we investigated the effects of EODF on expression levels of neurotrophic factors and antioxidants in the retina.

**Figure 6:**
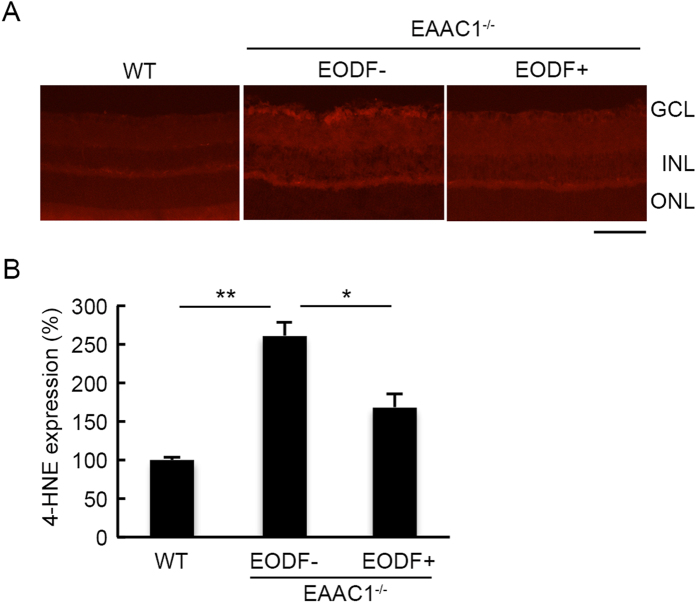
Effects of EODF on oxidative stress levels in the EAAC1^−/−^ mouse retina. (**A**) Representative images of the 4-HNE expression in the retina of mice at 12 W. Scale bar: 100 μm. (**B**) Quantitative analyses of (**A**). Data are normalized to the 4-HNE intensity at the GCL in control WT mice (100%). The data are presented as means ± SEM of six samples for each experiment. **P < 0.01, *P < 0.05.

Quantitative PCR analysis demonstrated that both BDNF and basic fibroblast growth factor (bFGF) expressions were significantly upregulated by EODF treatment although expressions of glial cell-derived neurotrophic factor (GDNF) and nerve growth factor (NGF) were unchanged (Fig. 5B). These data suggest that neuroprotection of EODF might be partially due to the upregulated neurotrophic factors. Moreover, catalase, a well-defined oxidative stress resistance gene, was found to be upregulated by EODF (Fig. 5B), indicating that oxidative stress in the retina might be suppressed by EODF. To confirm this point, we carried out immunohistochemical analyses of 4-hydroxy-2-nonenal (4-HNE), which represents oxidative stress levels, in the retinas of WT and EAAC1^−/−^ mice treated with or without EODF. As previously reported^10^, a significant increase of 4-HNE intensity was observed in EAAC1^−/−^ mice, but EODF treatment partially suppressed its expression levels (Fig. 6). Taken together, these results suggest that EODF prevents retinal degeneration in EAAC1^−/−^ mice by upregulating expressions of neurotrophic factors and reducing oxidative stress levels in the retina.

## Discussion

Herein, we demonstrate that EODF attenuates NTG-like retinal degeneration in both the histological and functional aspects. Consistent with the previous findings that EODF affects multiple signaling pathways^12^, its benefits in EAAC1^−/−^ retina are likely to be due to more than one single mechanism. At first, EODF significantly increased concentration of β-HB in the blood (Fig. 1B). β-HB, one of three ketone bodies (KBs), is an important metabolic substrate produced by the liver under conditions of fasting, caloric restriction, and intake of high-fat and low-carbohydrate diets such as the KD^27^. When glucose is in short supply, KBs serve as the brain’s principal alternative fuel. Recent studies have shown that KBs are not only substrates for fuel, but may also exert a cellular protective effect via an antioxidant mechanism^15^,^28^,^29^,^30^,^31^. Moreover, it has been shown that β-HB blocks the activity of a class of enzymes called histone deacetylases and thereby helps cells resist oxidative stress^13^. Oxidative stress is an important risk factor in human glaucoma^32^, and studies with DBA/2J mice demonstrated that the antioxidant α–lipoic acid protects RGCs in the glaucomatous retina^33^. In the present study, increased histone acetylation was found in the EODF retina, which might alter various gene expressions and contribute to the increased antioxidant gene, reduced oxidative stress levels, and RGC neuroprotection in EAAC1^−/−^ retina. These results are consistent with our previous reports that valproic acid, an effective HDAC inhibitor^34^,^35^, have neuroprotective effects on the retina of GLAST^−/−^ mice, the other mouse model of NTG, and prevents NMDA-induced RGC death^9^,^21^.

β-HB is normally present at very low concentrations in human blood (0.05 mM), and effects have been made to increase its concentrations safely for therapeutic purposes^36^. There are three available approaches to increase blood levels of β-HB currently. One is KD, which has long been used for the treatment of epilepsy although the mechanism responsible for the beneficial effect is not known^36^. However, the very high-fat, very low-carbohydrate, low-protein KD exerts adverse effects such as rises in plasma low density lipoprotein cholesterol, uric acid, and free fatty acids. Occasionally, the KD may be associated with an increased incidence of nephrolithiasis and other serious complications^37^. The other two available approaches to elevate blood levels of β-HB are two types of chemicals. One is esters of β-HB and the other is small synthetic, digestible KB polymers (including dimers). Since β-HB could be an effective therapy for a variety of diseases such as epilepsy, Alzheimer’s and Parkinson’s disease^15^,^28^,^29^,^30^,^31^, approaches to increase β-HB concentration safely are worth pursuing. For example, some caloric restriction mimetic such as resveratrol and sulforaphane might have similar benefits on neuroprotection^38^,^39^.

Recent studies have shown that β-HB ameliorates corneal epithelial erosion and superficial punctate keratopath, which are both hallmarks of dry eye disease, in a rat model^40^,^41^. More recently, 1% β-HB ophthalmic solution significantly improved both corneal and conjunctival symptoms in patients with an impaired capacity for tear secretion^42^. Our present findings suggest a possibility that EODF may improve corneal lesion. In addition, it will be interesting to investigate whether the β-HB solution eyedrop has neuroprotective effects on EAAC1^−/−^ mice.

In summary, we have demonstrated that EODF exerts neuroprotective effects and ameliorates visual impairment in a mouse model of NTG. Our findings raise interesting possibilities that EODF is beneficial for glaucoma patients.

## Methods

### Mice

Experiments were performed using C57BL/6J (WT) mice and EAAC1^−/−^ mice (Miltenyi Biotec GmbH, Bergisch Gladbach, Germany)^5^,^7^,^8^,^10^ on a C57BL/6J background in accordance with the Tokyo Metropolitan Institute of Medical Science Guidelines for the Care and Use of Animals. EAAC1^−/−^ mice were divided into two groups, a control group that had continual access to food (*n* = 7), and an EODF group that was provided food on alternate days (*n* = 7). Both groups were given ad libitum access to water. All experiments were approved by the Tokyo Metropolitan Institute of Medical Science.

### β-HB measurement

To evaluate the effects of EODF, a drop of blood was obtained by cutting the tail of the mouse every 24 h, namely right before the starting of the fasting regime and 24 h later when the fasting regime ends. And β-HB concentrations in the blood were measured by Precision Xceed (Abbott Laboratories, Lake Bluff, IL, USA) according to the manufacturer’s instructions for 7 days (Fig. 1A).

### Histopathology and quantification

Mice were anesthetized with an intraperitoneal injection of sodium pentobarbital and perfused transcardially with saline, followed by 2% paraformaldehyde in 0.1 M phosphate buffer containing 15% picric acid. Eyes were removed, postfixed and processed as previously reported^6^,^43^. Briefly, eyes were embedded in paraffin wax and sagittal sections through the optic nerve at the thickness of 7 μm were collected and stained with hematoxylin and eosin (H&E). The number of neurons in the GCL of the retina was counted from one ora serrata through the optic nerve to the other ora serrata. In the same section, the thickness of the IRL (between the internal limiting membrane and the interface of the outer plexiform layer with the outer nuclear layer) was measured at the distance 500 μm from the optic nerve. Three measurements were made for averaging in each retinal section. For immunohistochemistry staining, mice were fixed in the same fixative as for H&E staining, eyes were then removed, postfixed and immersed in 30% sucrose in 100 mM phosphate buffer (pH 7.4). Retinal cryostat sections of 14 μm thickness were prepared and examined by immunostaining using a 4-HNE mouse monoclonal antibody (0.2 μg/mL; Japan Institute for the Control of Aging, Shizuoka, Japan). The sections were then incubated with Cy-3-conjugated donkey anti-mouse IgG (Jackson ImmunoResearch, West Grove, PA). The image of the GCL was selected specifically and only the intensity of 4-HNE at the GCL was analyzed using NIH Image (ImageJ 1.50c4)^9^,^10^.

### Imaging acquisition of SD-OCT

SD-OCT (RS-3000; Nidek, Aichi, Japan) examinations were performed at 12 W on WT and EAAC1^−/−^ mice as previously reported^7^,^8^,^9^,^10^,^20^,^21^,^22^. Briefly, a 60-D adapter lens was placed on the objective lens of the Multiline OCT to focus on the mouse retina. All the images were location matched, scanning vertically through the center of the optic nerve head at a 3 disc diameter length above the optic nerve head. The average thickness of the GCC (between the internal limiting membrane and the interface of the inner plexiform layer and the inner nuclear layer) was measured. In this study, the maximum number of B-scans set by the manufacturer (50 for line scans) was used for averaging.

### mfERG

Mice were anesthetized by intraperitoneal injection of 87.5 mg/kg sodium pentobarbital. The pupils were dilated with 0.5% phenylephrine hydrochloride and 0.5% tropicamide. mfERGs were recorded using a VERIS 6.0 system (Electro-Diagnostic Imaging, Redwood City, CA, USA). The visual stimulus consisted of seven hexagonal areas scaled with eccentricity. The stimulus array was displayed on a high-resolution black and white monitor driven at a frame rate of 100 Hz. The 2 K component, which is impaired in patients with glaucoma, was analyzed as previously reported^5^,^6^,^7^,^8^,^9^,^10^,^21^,^23^,^24^.

### IOP measurement

IOP was measured by a commercial rebound tonometer (TonoLab; Colonial Medical Supply, Franconia, NH, USA) in anesthetized mice as reported previously^6^,^7^,^8^,^10^. To minimize variation, the data were collected during a time window of 4–6 min after injection of the anesthetic, during which IOP plateaus. IOP was measured at 12 W on EAAC1^−/−^ mice. Since the 24 h IOP pattern in mouse eyes is biphasic, with IOP being highest at around 21:00^44^, we examined IOP between 20:00 and 23:00.

### Retrograde labeling

Mice were deeply anesthetized with isoflurane (Intervet, Tokyo, Japan), placed on a stereotaxic frame, and injected with 2 μL of 2% FG (Fluorochrome LLC, Denver, CO, USA) dissolved in phosphate-buffered saline (PBS) into the superior colliculus^6^,^21^,^45^,^46^. Ten days after FG application, mice were anesthetized, eyes were enucleated, and retinas were isolated for whole mount preparation. Retinas were fixed in 4% paraformaldehyde in 0.1 M PBS solution for 20 min, mounted on a glass slide with a mounting medium (Vectashield; Vector Laboratories, Burlingame, CA, USA), and the RGC density was examined with a fluorescent microscope. The excitation and emission wavelengths for FG were 323 nm and 620 nm, respectively. Three standard areas (0.04 mm^2^) of each retina at a point 0.1 mm from the optic disc were randomly chosen. FG-labeled cells were manually counted, and the mean number of RGCs per square millimeter was calculated^45^.

### Quantitative real-time PCR

Total RNA of the retina was extracted with NucleoSpin^®^ RNA (Macherey-Nagel, Düren, Germany), and reverse-transcribed with Revertra Ace (Toyobo, Osaka, Japan) to obtain cDNA. Quantitative real-time PCR was performed using the MyiQ^TM^ single-color real-time PCR system (Bio Rad, Hercules, CA, USA) with Thunderbird SYBR qPCR MiX (Toyobo) as previously reported^47^. The primer sets used were as follows: BDNF forward primer 5′-ATG CCG CAA ACA TGT CTA TGA G-3′, BDNF reverse primer 5′-TGA CCC ACT CGC TAA TAC TGT CA-3′; bFGF forward primer 5′-CAC CAG GCC ACT TCA AGG A-3′, bFGF reverse primer 5′-GAT GGA TGC GCA GGA AGA A-3′; GDNF forward primer 5′-GGC CTA CCT TGT CAC TTG TTA GC-3′, GDNF reverse primer 5′-GGC CTA CTT TGT CAC TTG TTA GC-3′; NGF forward primer 5′-CGA CTC CAA ACA CTG GAA CTC A-3′, NGF reverse primer 5′-GCC TGC TTC TCA TCT GTT GTC A-3′; catalase forward primer 5′-CCG ACC AGG GCA TCA AAA-3′, catalase reverse primer 5′-GAG GCC ATA ATC CGG ATC TTC-3′; and glyceraldehyde 3-phosphate dehydrogenase (GAPDH) forward primer 5′-TGC ACC ACC AAC TGC TTA G-3′, GAPDH reverse primer 5′-GGA TGC AGG GAT GAT GTT C-3′). Data were normalized to the level of GAPDH mRNA.

### Acid extraction of histones and immunoblot analysis

To investigate the effects of EODF on histone acetylation, retinas from WT mice treated with EODF for 7 days were isolated and histones were extracted as previously reported with slight modification^13^. Snap-frozen mouse retinas were ground and dissolved in 1 ml of lysis buffer (1 × PBS with 0.5% Triton, 2 mM PMSF, 0.02% NaN3, and 5 mM sodium butyrate). Lysates were mixed with agitation for 10 min at 4 °C, and then centrifuged at 500 g for 10 min at 4 °C. Precipitates were washed twice in 1 ml of lysis buffer, resuspended in 0.2 N HCL and incubated with agitation for overnight at 4 °C. Histones in supernatant were collected after centrifugation at 500 g for 10 min. Tris-base was added into the histone fraction to neutralize before sodium dodecyl sulfate-polyacrylamide gel electrophoresis (SDS-PAGE). Immunoblotting was carried out as previously reported^48^. Samples were separated on a SDS-PAGE and subsequently transferred to an Immobilon-P filter (Millipore, Milan, Italy). Membranes were incubated with antibodies against acetyl-histone H3_K9_ (1:2000, Cell signaling, Danvers, MA, USA), acetyl-histone H3_K18_ (1:2000, Cell signaling) and histone H3 (1:2000, Cell signaling).

### Statistics

Data are presented as mean ± SEM. The Student’s *t*-test was used for statistical analyses and results were considered to be significant at *P* < 0.05.

## Additional Information

**How to cite this article**: Guo, X. *et al*. Caloric restriction promotes cell survival in a mouse model of normal tension glaucoma. *Sci. Rep.* **6**, 33950; doi: 10.1038/srep33950 (2016).
